# Complementary and alternative therapies in the treatment of primary dysmenorrhea

**DOI:** 10.3389/frph.2025.1730164

**Published:** 2026-01-13

**Authors:** Wenyi Ma, Linyan Qiu, Yanfeng Dong, Huifang Zhou

**Affiliations:** 1 Nanjing University of Chinese Medicine, Nanjing, Jiangsu, China; 2 Affiliated Hospital of Nanjing University of Chinese Medicine (Jiangsu Province Hospital of Traditional Chinese Medicine), Nanjing, Jiangsu, China

**Keywords:** complementary and alternative therapy, gynecology, primary dysmenorrhea, therapie, women's health

## Abstract

Primary dysmenorrhea is a common gynecological condition among women. Complementary and alternative medicine (CAM) has been used for the treatment of primary dysmenorrhea for centuries. These therapies lack the drawbacks associated with traditional symptomatic medications, such as non-steroidal anti-inflammatory drugs (NSAIDs) and oral contraceptives, which can increase the risk of adverse effects like mild neurological symptoms (headaches, drowsiness, dizziness) and gastrointestinal symptoms (nausea, indigestion). This article reviews current CAM strategies for treating primary dysmenorrhea, including vitamins, herbal supplements, acupuncture, exercise, transcutaneous electrical nerve stimulation (TENS), acupressure, and aromatherapy. The article analyzes the benefits and potential mechanisms of these therapies, aiming to provide practitioners with the most commonly used and widely recommended CAM methods. Finally, the review highlights future directions for dysmenorrhea treatment, including ongoing research and potential new therapies. The goal of this review is to provide a brief summary of the current literature on the most commonly used CAM approaches for patients with primary dysmenorrhea, as a comprehensive approach to managing primary dysmenorrhea and improving quality of life.

## 1 Introduction

**Figure 1:**
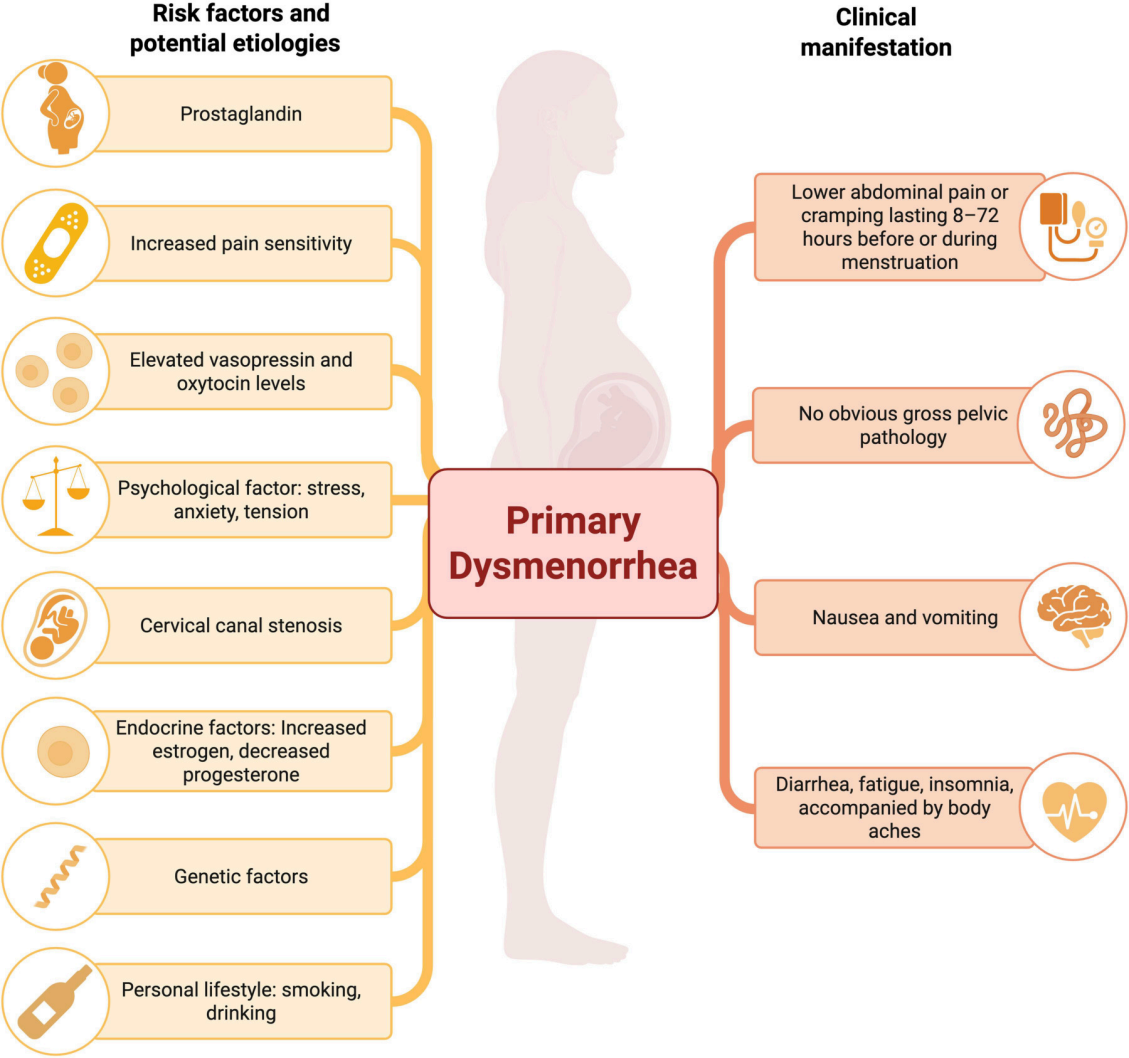
The clinical manifestations and pathogenesis of primary dysmenorrhea.

Primary dysmenorrhea is a menstrual disorder characterized by pain that begins shortly before or at the onset of menstruation and can last up to 72 h. The pain is located in the suprapubic area and may radiate to the upper thighs, lower back, or both. It commonly occurs in adolescents and young adult women (ages 16–25) and is one of the leading causes of pelvic pain. Nonsteroidal anti-inflammatory drugs (NSAIDs) are the first-line analgesic therapy for dysmenorrhea, as the pathophysiology of the condition involves the release of prostaglandins. NSAIDs alleviate pain by inhibiting cyclooxygenase (COX-1 and COX-2), which prevents the production of prostaglandins (1). Another pharmacological treatment for primary dysmenorrhea involves hormone-based contraception. For women who consent to hormonal treatment and seek contraception, hormonal contraception is a good option in addition to dysmenorrhea management. Estrogen inhibits the release of FSH, ultimately preventing ovulation. Progesterone causes the endometrium to thin and cervical mucus to thicken, preventing sperm penetration. A thinner endometrium contains less arachidonic acid, which is used for prostaglandin synthesis, thus reducing menstrual blood flow and uterine contractions (2). Figure 1 illustrates the clinical manifestations and pathogenesis of primary dysmenorrhea.

Women with dysmenorrhea have a range of treatment options, including nonsteroidal anti-inflammatory drugs (NSAIDs), oral contraceptive pills (OCPs), COX-2 (cyclooxygenase-2) specific inhibitors, and complementary and alternative medicine (CAM). The goal of treatment is to achieve adequate pain relief and symptom management. Two Cochrane reviews have highlighted the effectiveness of conventional treatments (NSAIDs and OCPs). NSAIDs have been found to be more effective than placebo in relieving pain in women with primary dysmenorrhea, but they increase the risk of adverse effects, such as mild neurological symptoms (headache, drowsiness, dizziness) and gastrointestinal symptoms (nausea, indigestion). COX-2-specific inhibitors are effective for dysmenorrhea, but due to concerns about cardiovascular and heart protection safety, these drugs have been discontinued in many countries (1). Combined hormonal contraceptives are effective in treating dysmenorrhea in approximately 70%–80% of women. However, when combined OCP is used, there is an additional deep vein thrombosis (DVT) in every 1,000 women, and a higher risk is observed in users aged 6–12 months before use and over 40 years old (3). In addition to the known thrombotic effect of estrogen, the type of progesterone may also affect the risk of DVT, although the data are limited and contradictory (4–8). Not only that, adverse reactions related to estrogen may include nausea, vomiting, headache, breast tenderness, and weight changes; Adverse reactions related to progesterone may include acne, weight gain, increased hair growth, and depression (2).

**Figure 2:**
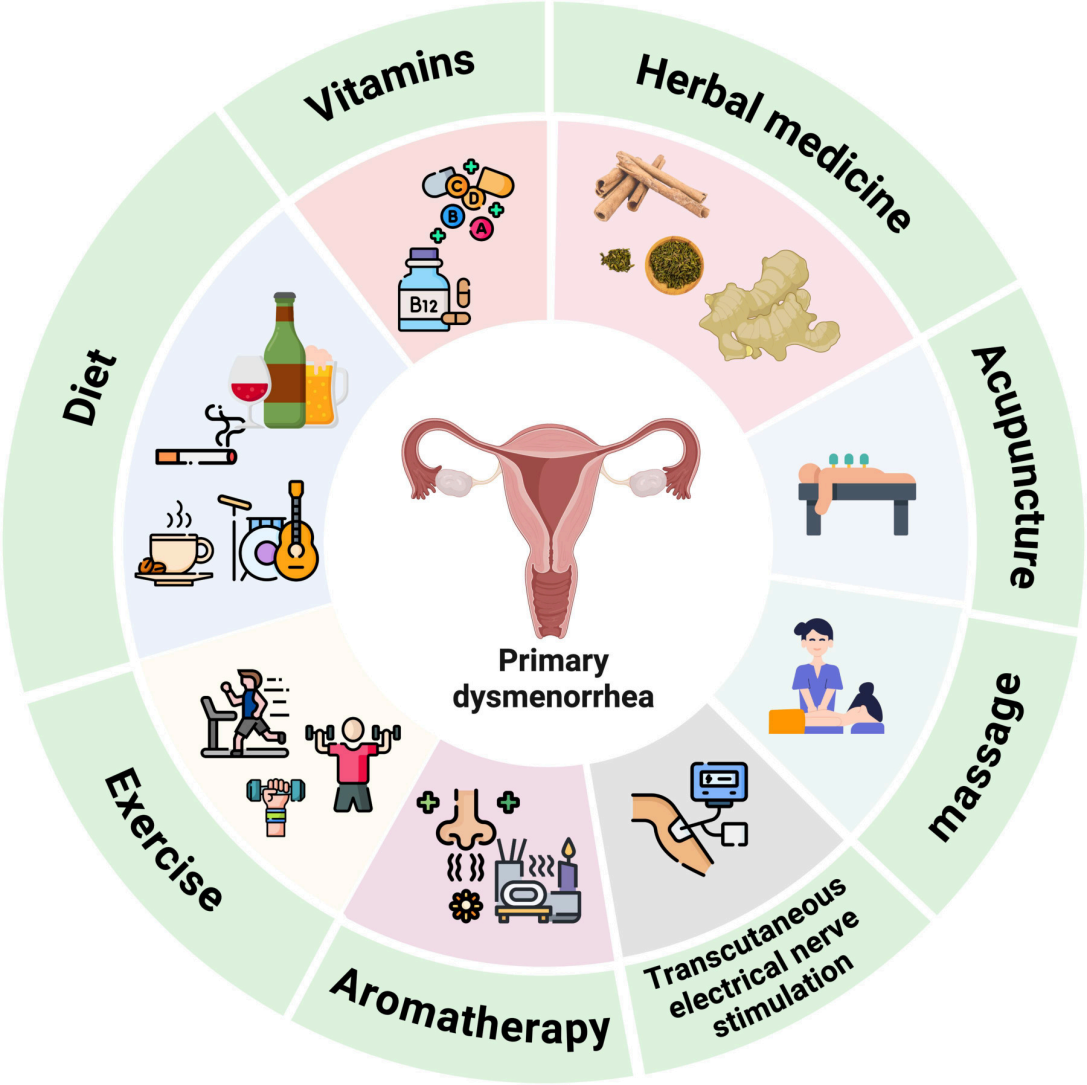
Complementary and alternative medicine.

Complementary and alternative medicine (CAM) is defined as a diverse group of medical systems, practices, and products that are not currently considered part of conventional medicine (3). “Complementary medicine” refers to non-mainstream approaches used with standard treatments, while “alternative medicine” replaces conventional care. These categories sometimes overlap and can shift over time. CAM for primary dysmenorrhea has a long history. Many women use CAM therapies alongside approved medications due to the drawbacks of traditional drugs. This review covers the role and application of complementary and alternative therapies such as diet, exercise, aromatherapy, massage, dietary supplements, and transcutaneous electrical nerve stimulation (TENS). These methods, rooted in Traditional Chinese Medicine, broaden women's treatment options and support overall health. Figure 2 summarizes the various complementary and alternative medicine approaches for primary dysmenorrhea.

CAM is widely used by patients with primary dysmenorrhea. Patients may not always tell their doctors about CAM use, while some seek professional advice. Doctors often have limited CAM knowledge and may minimize its role. This review briefly summarizes current literature on the most common CAM modalities for primary dysmenorrhea management. Although it does not cover every aspect of CAM, this review discusses the approaches shown to be most effective in clinical trials. Patients and providers are encouraged to use CAM together with pharmacological treatments to improve symptoms and quality of life.

## 2 Complementary and alternative medicine (CAM)

### 2.1 Ingestible substances

#### 2.1.1 Vitamins

The pathogenesis of dysmenorrhea is largely attributed to the excessive release of prostaglandins (PG), which leads to excessive uterine contractions, reduced uterine blood flow, and nerve hypersensitivity, thereby triggering dysmenorrhea. The synthesis of PG is limited by the availability of the free fatty acid precursor, arachidonic acid, which is regulated by cyclic adenosine monophosphate (cAMP). Through cAMP, the production of PG can be stimulated by substances such as adrenaline, peptide hormones, and steroid hormones, as well as by mechanical stimuli and tissue injury. Arachidonic acid is extracted from phospholipids by the lysosomal enzyme phospholipase A2. The large release of prostaglandins results in excessive uterine contractions, reduced uterine blood flow, and nerve hypersensitivity, all of which contribute to dysmenorrhea (4). Nonsteroidal anti-inflammatory drugs (NSAIDs) alleviate pain through a mechanism that involves the production of prostaglandins. Interestingly, many vitamins, such as vitamin D and vitamin E, are believed to participate in the molecular pathways of prostaglandins (5–7).

##### 2.1.1.1 Vitamin D

Vitamin D can reduce the production of prostaglandins in uterine tissues through several pathways. Firstly, vitamin D can decrease the expression of prostaglandin receptors, thereby inhibiting prostaglandin-mediated functional responses. Secondly, vitamin D accelerates the degradation of prostaglandins by enhancing the activity of 15-dehydrogenase. Finally, vitamin D reduces the expression of cyclooxygenase-2 (COX-2), similar to the mechanism of nonsteroidal anti-inflammatory drugs (NSAIDs). Additionally, by regulating the upstream transcription factor nuclear factor-kappa B (NF-κB), vitamin D significantly reduces the inflammatory cytokine cascade induced by interleukin (IL)-1β, IL-6, or tumor necrosis factor-alpha (TNF-α), which includes IL-8, PGE2, PGF2, and NF-κB activation (8); AAKT; mitogen-activated protein kinase (MAPK); as well as Janus kinase/signal transducer and activator of transcription 3 (JAK/STAT3) (9).The excessive uterine contractions involved in dysmenorrhea may be related to the activation of myosin light chain kinase induced by calcium ion influx (10). In *ex vivo* models, vitamin D alleviated contractions and calcium release associated with L-type calcium channels, suggesting a potential mechanism for its role in dysmenorrhea.

Studies have shown that when vitamin D is consumed at a dose greater than 50,000 IU per week over a period of more than 70 days, pain relief becomes more pronounced (11). Bahrami A et al. (12) Conducted a 9-week follow-up study of 897 girls living in Mashhad and Sabzevar, Iran, and found that high-dose vitamin D supplementation could reduce the incidence of dysmenorrhea, with positive effects on both the physical and psychological symptoms of dysmenorrhea.

##### 2.1.1.2 Vitamin E

Vitamin E plays a key role in limiting the production of prostaglandins. Since the arachidonic acid pathway is initiated by the oxidation of membrane fatty acids, Vitamin E can limit the production of arachidonic acid, thus preventing its conversion into prostaglandins that induce pain. In addition to inhibiting enzymes in the arachidonic acid pathway, Vitamin E also has antioxidant activity. Due to its significant antioxidant effects, Vitamin E serves as the first line of defense against membrane phospholipid peroxidation. There is evidence regarding the positive impact of Vitamin E in alleviating primary dysmenorrhea. In a study by Ziaei et al., 100 IU of Vitamin E was used daily for 5 days in 2 cycles, or 200 IU of Vitamin E was used daily for 2 cycles.

In Moslemi et al.'s study, 400 IU of Vitamin E was used for the first 3 days of the two cycles (13), while in a study by Kashanian et al., a prescription of 400 IU was used for the first two days of 4 cycles. All studies showed a significant reduction in pain severity in the Vitamin E intervention groups (14).

Evidence supporting the efficacy of vitamin supplements in alleviating dysmenorrhea pain is considered low quality, with numerous limitations including small sample sizes, lack of methodological reporting, and inconsistencies (15). Although data supporting supplement effectiveness is limited, most supplements pose minimal risk to patients and should be reviewed with healthcare providers to ensure no interactions with other prescribed medications. It is important to note that vitamin supplementation requires appropriate dosing. Excessive supplementation may increase adverse reactions such as liver damage, elevated intracranial pressure, bone pain, coagulation disorders, and peripheral neuropathy (numbness in hands and feet).

#### 2.1.2 Herbal supplements

In recent years, herbs have commonly been used to treat primary dysmenorrhea. In traditional Chinese medicine, dysmenorrhea is believed to be caused by the stagnation of cold and dampness in the uterus due to drinking cold beverages, being exposed to rain, or coming into contact with water. Blood coagulates when exposed to cold, leading to a lack of smooth flow in the uterus, causing stagnation and resulting in pain. Therefore, the treatment principle for dysmenorrhea should focus on warming the meridians, dispersing the cold, and removing dampness. As commonly used natural products, cinnamon (Cinnamomum zeylanicum), fennel (Foeniculum vulgare), and ginger (Zingiber officinale) all warm the meridians, disperse cold, and remove dampness.

##### 2.1.2.1 Cinnamon

Cinnamon is an aromatic spice that has been used to treat various inflammatory and chronic diseases. It has anti-spasmodic and analgesic effects, and despite its unpleasant taste, it is considered an acceptable herb for treating dysmenorrhea. The main component of cinnamon is cinnamaldehyde, and its essential oil contains 55%–57% cinnamaldehyde and 5%–18% eugenol. It has been reported that **Cinnamomum zeylanicum** exhibits antispasmodic effects. Eugenol can also inhibit the biosynthesis of prostaglandins and affect inflammation (16). Therefore, cinnamon is thought to exert its effects by inhibiting the prostanoid system involved in PGE2 production (17). Additionally, cinnamon can reduce uterine contraction force by inhibiting the influx of Ca^2+^ (18, 19). Research has shown that the components of cinnamon reduce intracellular calcium release by inhibiting L-type Ca^2+^ channels and calcium release from the sarcoplasmic reticulum (SR), exerting a vasodilatory effect on rat aortic smooth muscle cells.

Jahangirifar M et al (20). demonstrated that using 3 grams of cinnamon daily within a safe range significantly alleviates the severity of dysmenorrhea. Furthermore, their study found that taking 420 mg of cinnamon three times a day significantly reduced the severity of pain, nausea, vomiting, and bleeding compared to a placebo (20). Overall, cinnamon appears to be an effective remedy for treating primary dysmenorrhea, although it may not be equivalent to non-steroidal anti-inflammatory drugs (NSAIDs) (21). It is important to note that coumarin, a compound found in cinnamon, possesses anticoagulant properties. Individuals with bleeding disorders or those taking anticoagulant medications such as warfarin or aspirin should be aware that consuming large amounts of cinnamon can significantly increase the risk of bleeding. Additionally, excessive cinnamon powder may irritate the gastrointestinal tract, causing discomfort. Direct inhalation of cinnamon powder can irritate the lungs, leading to coughing and a sensation of suffocation, posing particular danger to asthma sufferers.

##### 2.1.2.2 Ginger

Ginger helps alleviate pain associated with dysmenorrhea, as well as gastrointestinal symptoms such as nausea and vomiting. Ginger is rich in non-volatile pungent compounds, such as various types of gingerols, shogaols, zingerone, and paradol. It is nutritionally rich and has significant pharmacological effects, including anti-inflammatory, antimicrobial, antitumor, anticoagulant, neuroprotective, and reproductive system protective properties (22–27).The accumulation of various substances such as prostaglandins, chemokines, cytokines, growth factors, and oxytocin can induce uterine hypercontraction and vasoconstriction, which are the main mechanisms of menstrual pain in primary dysmenorrhea. Gingerols and ginger diketones in ginger have anti-inflammatory properties and can inhibit leukotriene and prostaglandin production by inhibiting COX activity (28). Moreover, ginger compounds can interact with the lipoxygenase pathway to reduce the activity of 5-lipoxygenase. These compounds affect the synthesis of eicosanoids derived from arachidonic acid, which is believed to produce eicosanoids, including prostaglandin E2 and thromboxane, potentially causing inflammation and acting as platelet aggregation inducers (29–32).The effect of ginger on cyclooxygenase activity leads to a reduction in the production of prostaglandins and thromboxanes through the cyclooxygenase pathway, which has been confirmed in several studies. Therefore, ginger can activate endorphin receptors and act as a coagulation enzyme inhibitor, potentially effective in relieving pain related to dysmenorrhea.TRPV1 channel proteins, which are involved in pain sensitization in primary dysmenorrhea (PD), are activated by various ligands, inflammatory mediators (such as arachidonic acid metabolites), and tissue damage stimulants. Ginger can mediate the ERK1/2/NF-κB signaling pathway to regulate TRP ion channels and treat dysmenorrhea (33). Thus, ginger can alleviate primary dysmenorrhea through multiple mechanisms.

A systematic review and meta-analysis by Daily JW et al. provided suggestive evidence of the effectiveness of ginger in treating primary dysmenorrhea. The study clearly showed that ginger (750–2000 mg daily during the first 3–4 days of the menstrual cycle) is a promising potential treatment for pain and discomfort related to primary dysmenorrhea. The results from Xu Y et al. (34) also indicated that cinnamon effectively reduced the intensity and duration of pain, consistent with Daily JW's (35)findings. However, there is currently a lack of studies comparing ginger with other drugs that are commonly used to treat dysmenorrhea. Although some studies have found that ginger and non-steroidal anti-inflammatory drugs (NSAIDs) are equally effective in reducing pain severity, these results should be viewed with caution due to the limited number of studies, low consistency in methods, and high heterogeneity in trials (36). Additionally, ginger should be used in moderation, with no more than 10–15 g of fresh ginger or 1–3 g of dried ginger powder daily. Improper use may increase the risk of gastrointestinal irritation and bleeding.

##### 2.1.2.3 Fennel

Fennel has been widely used as a global herbal remedy. The main components of fennel oil are fenchone (60%–80%) and anethole (10%–30%), along with other compounds such as estradiol, α-pinene, flavonoids, coumarins, and glycosides (37). Fennel alleviates dysmenorrhea by reducing prostaglandin levels in the blood (38). The Drug Information Center recommends taking 1–1.5 cups of fennel powder daily to reduce dysmenorrhea. Its anti-spasmodic effect on contractions caused by oxytocin and PGE2 has been confirmed in uterine tissues dissected from mice (39). A study on the association between fennel and infant colic showed that fennel seed oil emulsion could reduce the intensity of spasms in infants (40).

Research by Nahid et al. (41) found that after two months of treatment with a combination of fennel, celery, and saffron, the pain intensity score decreased from 5.3 to 3.0, and further decreased to 0.5 after three months. A systematic analysis by Xu Y et al. (34) Also confirmed that fennel significantly reduces the intensity of pain associated with primary dysmenorrhea. Therefore, fennel is proven to significantly alleviate pain associated with primary dysmenorrhea. However, some participants in the studies complained that the smell of fennel could induce nausea, so in future treatments, fennel may be better used in tablet or capsule form to avoid this issue. It is worth noting that some individuals consume large quantities of fennel tea daily over extended periods for purported benefits such as “weight loss,” “relieving constipation,” or “regulating digestion.” This practice may lead to hormonal imbalances or other unknown chronic health risks.

#### 2.1.3 Diet

Diet and nutrition are crucial for maintaining overall health, including that of women. Gene-nutrient interactions play a significant role in health management and disease prevention. Previous studies have analyzed the potential impact of certain foods on dysmenorrhea, highlighting the protective effects of increasing the consumption of fruits, vegetables, fish, and dairy products on dysmenorrhea, while foods such as coffee, fats (42), and sugary foods are considered susceptible to primary dysmenorrhea (43, 44). The active ingredients in pomegranate juice can reduce uterine contractions and treat uterine tension disorders, with the potential to prevent preterm birth and dysmenorrhea. However, further research is needed to determine its mechanism of action (45). Honey and tryptophan capsules have been found to relieve the same level of pain in women with primary dysmenorrhea (46, 47). Moreover, honey has fewer side effects and pharmacological complications, so it is recommended for pain relief.

In fact, Barnard (48) found that women on a low-fat diet had a significantly reduced severity of primary dysmenorrhea. A Cochrane systematic review found that vitamins B1 and B6 and fish oil (omega-3 fatty acids) were more effective than placebos in reducing the severity of primary dysmenorrhea pain (49). In this study, the severity of dysmenorrhea in the diet and control groups was 7.14 ± 1.3 and 7.09 ± 1.4, respectively, before the treatment. After dietary treatment, the severity of dysmenorrhea in the diet group was significantly lower than that in the control group (control group: 6.74 ± 1.97, diet group: 5.15 ± 1.15). Research by Onieva-Zafra, MD et al. (50) found that women who ate fewer than two servings of fruit per day were more likely to experience dysmenorrhea. Similarly, women who enjoyed beans and ate them more than once a week were also more likely to experience dysmenorrhea. Compared to women with dysmenorrhea, those without dysmenorrhea had higher daily intakes of strawberries and olive oil, while women with dysmenorrhea who ate pickled ham at least once a week had slightly higher consumption and percentage. Zakaria IA et al. (51) confirmed that probiotics showed potential in reducing non-steroidal anti-inflammatory drug (NSAID) usage and improving pain and mental health. In a study conducted by Abu Helwa et al., skipping breakfast was identified as the strongest predictor of the severity of dysmenorrhea (43). Therefore, we can conclude that dietary interventions are an effective non-pharmacological treatment for young girls to alleviate the severity of primary dysmenorrhea symptoms. However, these studies have some limitations. The sample sizes are small, and thus, there is insufficient power to detect significant differences in outcomes such as inflammatory markers and the frequency of NSAID usage. The confounding effect of NSAID use was not considered, and although self-reports were used, they are susceptible to reporting bias. Table 1 summarizes the key clinical trials on ingestible substances for primary dysmenorrhea.

**Table 1:** Ingestible substances.

Therapy	Research type	Number	Time	Effect/result
Vitamin				
	Randomized controlled trial (RCT) (44)	60	2021	After the treatment with vitamin C and vitamin E, the severity of pelvic pain in the treatment group significantly decreased compared to the placebo group.
	Pre-post study (12)	897	2018	Primary dysmenorrhea patients who received nine types of high-dose vitamin D supplements experienced a reduction in pain severity.
	Pre-post study (46)	85	2023	Vitamin D can be a useful treatment option to reduce the severity of primary dysmenorrhea and limit the use of non-steroidal anti-inflammatory drugs (NSAIDs).
	Single-blind clinical trial (131)	Students aged 18–25	2019	Vitamin D, vitamin E, and ginger significantly reduced the severity of dysmenorrhea, with ginger having the most significant effect, followed by vitamin D and vitamin E.
	Double-blind, randomized, placebo-controlled trial (132)	112	2021	Supplementing vitamin D in women with primary dysmenorrhea and vitamin D deficiency can improve systemic symptoms, reduce pain intensity, decrease the number of pain days, and lower the need for painkillers.
	A double-blind, randomized clinical trial (133)	75	2018	Supplementing vitamin D in women with primary dysmenorrhea and vitamin D deficiency can improve systemic symptoms, reduce pain intensity, decrease the number of pain days, and lower the need for painkillers.
Herbal supplements				
Cinnamon				
	Randomized controlled trial (RCT)肉 (20)	58	2018	Cinnamon can reduce the intensity of primary dysmenorrhea. This aromatic spice is recommended for the management of primary dysmenorrhea.
Ginger				
	Randomized controlled trial (RCT) (33)	122	2014	Ginger is more effective than tolfenamic acid in alleviating primary dysmenorrhea pain.
	Randomized controlled trial (RCT) (131)	200	2019	Ginger is the most effective supplement among those taken. Vitamin D appears to be more effective than vitamin E in relieving dysmenorrhea.
	Randomized controlled trial (RCT) (134)	336	2018	Ginger is as effective as Novafen in relieving primary dysmenorrhea.
Fennel				
	Randomized controlled trial (RCT) (40)	110	2006	There is no significant difference in the level of pain relief between fennel and tolfenamic acid for dysmenorrhea.
	Randomized controlled trial (RCT) (135)	59	2013	Fennel, with a 2% reduction in primary dysmenorrhea pain relief, shows efficacy comparable to that of common NSAIDs like tolfenamic acid.
Diet				
	Cross-sectional study (136)	660	2022	To overcome severe dysmenorrhea, women should increase their intake of fish and fresh fruits, as well as drink water.
	Randomized controlled trial (RCT) (137)	56	2017	Honey and tolfenamic acid capsules relieved the same amount of pain in women with primary dysmenorrhea.
	Cross-sectional study (138)	59	2024	Women with menstrual disorders tend to consume more high-sugar foods and beverages, leading to inadequate nutritional intake.
	Randomized controlled trial (RCT) (139)	86	2019	Compared to the placebo, using propolis for two months can reduce primary dysmenorrhea in the first and second months after use, with no adverse effects.
	Randomized controlled trial (RCT) (140)	70	2018	Dietary therapy is effective in reducing pain in female college students with primary dysmenorrhea.
	Randomized controlled trial (RCT) (50)	72	2024	The tested oral probiotic improved mental health and may have reduced the use of non-steroidal anti-inflammatory drugs; however, there were no significant changes in inflammatory markers.

### 2.2 Non-ingestion intervention

#### 2.2.1 Iranscutaneous electrical nerve stimulation (TENS)

Transcutaneous Electrical Nerve Stimulation (TENS) delivers pulsed electrical currents across the surface of the skin to stimulate peripheral nerves, thereby alleviating pain. The pain gate theory, proposed in 1965, suggests that large-diameter (Aβ) afferent fibers (which carry vibration, touch, etc.) reduce pain perception by inhibiting nociceptive activity in the dorsal horn of the spinal cord (52). TENS induces analgesia by stimulating large-diameter fibers. TENS produces pain relief at the spinal cord segment level and is considered to have an additional spinal segment effect. It can reduce dorsal horn neuronal sensitization (53) caused by inflammation, alter the levels of neurotransmitters such as gamma-aminobutyric acid (GABA) and glycine, which are believed to participate in inhibiting pain transmission (54), and regulate the activity of glial cells that provide support and surround neurons in the spinal cord (55). Furthermore, TENS may influence endogenous analgesia. The descending activity transmitted through the periaqueductal gray (PAG) and rostral ventromedial medulla (RVM) in the brainstem may exert inhibitory effects at the segmental level (56). This PAG-RVM relay segmental inhibition is partly mediated through opioid pathways (56, 57).

Clinical studies by Guy M et al. (58) have confirmed that, compared to the control group, the TENS group of primary dysmenorrhea patients experienced a 54% reduction in pain. Clinical reviews by Han S et al. (59) also confirmed that both high-frequency and low-frequency TENS can alleviate pain compared to a placebo or no treatment. Therefore, TENS is highly effective in treating primary dysmenorrhea. However, to achieve optimal TENS efficiency, the most important parameter is the intensity of the current. The intensity should continuously increase throughout the treatment to maintain a strong sensation and avoid habituation. Due to the risk of bias, the certainty of evidence is reduced. Future randomized controlled trials (RCTs) should focus more on secondary outcomes such as the need for additional analgesics, limitations on daily activities, or health-related quality of life, and ensure a low risk of bias.

#### 2.2.2 Acupuncture

Acupuncture has been practiced for thousands of years as a complementary and alternative therapy for treating primary dysmenorrhea. Previous acupuncture studies on dysmenorrhea have also found pain relief, which supports these research findings. The improvement of dysmenorrhea symptoms in primary dysmenorrhea patients may be attributed to acupuncture's central and peripheral analgesic effects, neuroendocrine activity, and the expression of related receptors.

First, from a central perspective, acupuncture stimulation of acupoints has been shown to trigger the release of endorphins in the periaqueductal gray (PAG), arcuate nucleus, and caudate nucleus (60). These structures send projections to the dorsal horn of the spinal cord via the dorsal column. Wang Y et al. (61) found in a comparison between true acupuncture and sham acupuncture that there were changes in resting-state functional connectivity (FC) between the rostral anterior cingulate cortex (rACC) and the left central anterior gyrus after treatment. Baseline rACC-left central anterior gyrus FC was negatively correlated with short-term pain relief, while changes in rACC-left central anterior gyrus FC were positively correlated with short-term pain relief after acupuncture treatment. These findings support the importance of rACC-left central anterior gyrus resting-state FC in modulating primary dysmenorrhea (PDM) pain intensity through acupuncture, which may clarify the central mechanism of acupuncture in treating PDM.

From a peripheral perspective, Pomeranz et al. proposed that acupuncture stimulation activates A-*δ* and C afferent fibers in muscles (62). During acupuncture at points such as SP6, SP8, and Ren 4, signals are transmitted to the spinal cord and then relayed to the midbrain via afferent pathways. It has been reported that acupuncture can stimulate nerves in local tissues, leading to the release of neuropeptides, which cause vasodilation and increase blood circulation in the target area. This increase in blood circulation dilutes pro-inflammatory molecules such as prostaglandins, kinins, and histamine (which trigger pain) within the vessels; enhances tissue oxygenation; promotes drainage and a “cleansing” effect; and clears tissue debris and byproducts of tissue damage.

Animal studies have shown that acupuncture may help alleviate dysmenorrhea symptoms by regulating the neuroendocrine activity of the hypothalamic-pituitary-ovarian (HPO) axis and the expression of related receptors (63). The role of vascular changes in the causality of dysmenorrhea is well-documented. Previous research has suggested that dysmenorrhea is caused by reduced blood flow due to excessive uterine activity. This occurs when uterine contractions decrease blood flow, which can be alleviated by increasing blood circulation and using antispasmodics (64). Acupuncture has been shown to increase nitric oxide levels, which relax smooth muscles and help inhibit uterine contractions, thereby reducing spasms and other dysmenorrhea symptoms (65). Acupuncture may also alter the metabolism of substrates involved in the ascending facilitation pathways, such as N-methyl-D-aspartate (NMDA) receptors (66), substance *P* (67), and interleukin-1 (68), as well as the descending inhibitory pain pathways, including endogenous opioids (69), serotonin (70), and norepinephrine (71).

These findings suggest that acupuncture is a safe and acceptable treatment for women. Acupuncture has long been used to manage various types of pain, with substantial evidence supporting its effectiveness in pain management (72). The World Health Organization (WHO) recommends acupuncture for several conditions, including primary dysmenorrhea (PD) (73). Acupuncture actively regulates kidney-chong-ren-intrauterine hormone levels, affects the neuroendocrine system of patients, increases prostaglandin E2 (PGE2) levels, decreases prostaglandin F2*α* (PGF2*α*) levels, and alleviates symptoms and pain in PD patients (74). Three high-quality randomized controlled trials (RCTs) indicate that those receiving acupuncture experience less pain than those receiving placebo treatments (75–77). Cho et al. (78) found that acupuncture was significantly associated with pain reduction compared to drug treatments or herbal therapies. Furthermore, one study found no differences in the reduction of average visual analog scale (VAS) scores among different acupuncture points (77). However, due to the study design, it only demonstrated the effects of different acupuncture points on primary dysmenorrhea, and the overall impact of acupuncture therapy on the condition could not be excluded. Additionally, the relatively small number of participants in these studies might reduce statistical power. Therefore, the potential effects of acupuncture-related therapies need to be validated through large, well-designed trials with randomization, blinding, and control groups.

#### 2.2.3 Finger pressure

Shiatsu is defined as a form of needleless acupuncture, primarily aimed at stimulating energy channels called meridians and specific areas associated with particular organs by applying pressure (79). This technique involves applying pressure, and it has been shown to reduce muscle tension and promote relaxation in various areas of the body. Shiatsu is also known as acupressure, and research indicates that acupressure can alleviate menstrual symptoms (80), reduce the severity and duration of menstrual pain, as well as alleviate distress and anxiety. Additionally, it helps improve the quality of life and well-being of patients, providing psychological support and promoting self-care (81). As pointed out in the Korakaba theory, dysmenorrhea is an issue affecting comfort needs on four levels—physical, psychological, social-cultural, and environmental. Shiatsu not only alleviates pain through its sedative and analgesic effects but also enhances comfort. Existing evidence suggests that acupressure increases the release of endogenous opioids, such as endorphins, as well as neurotransmitters, including serotonin, by stimulating free nerve endings, which provide analgesic and sedative effects (82, 83). Most studies have also shown that it can improve pain intensity in primary dysmenorrhea. Moreover, it has been demonstrated that shiatsu can stimulate the secretion of endorphins, which are neurochemicals that relieve pain, as well as enhance blood circulation and oxygen supply, thus promoting relaxation.

Various clinical studies have examined the impact of acupressure on menstrual discomfort. Two studies conducted in Taiwan showed that acupressure at the Sanyinjiao acupoint significantly reduced dysmenorrhea pain and distress in adolescent girls during a 3- to 6-month follow-up period (84, 85). Another Taiwanese study involving 134 girls with dysmenorrhea found that after 6 months of follow-up, participants who received acupressure at the Hegu and Sanyinjiao acupoints showed significant reductions in menstrual pain, menstrual distress, and anxiety during menstruation (86). A study by Chen H M et al. (87) demonstrated the short-term, medium-term, and long-term effects of acupressure on relieving menstrual distress and confirmed that acupressure can be a safe, simple, non-pharmacological treatment for menstrual discomfort and low back pain. Thus, we can conclude that acupressure is an effective method for managing primary dysmenorrhea and, as a low-cost, easy-to-use, and non-pharmacological treatment, women can apply this method anywhere. However, there is a need for higher-quality randomized controlled trials with larger sample sizes to validate our conclusions. Future studies should explore acupoint interventions targeting other dysmenorrhea symptoms to provide more comprehensive care for dysmenorrhea patients. Additionally, to determine the effectiveness of acupressure for pain relief, it is crucial to appropriately adjust the stimulation dose, including the frequency, duration, and intensity of application.

#### 2.2.4 Aromatherapy

Multiple pieces of evidence suggest that aromatherapy is an effective intervention for reducing dysmenorrhea. Aromatherapy has historically been used for various purposes and forms, such as massage, inhalation, and bathing. Because these methods can be easily used in public by anyone, aromatherapy offers positive effects for users. It can enhance participants’ satisfaction, improve psychological stability (such as reducing depression, anxiety, and stress), and affect hormones, endocrine glands, and the circulatory system. Aromatherapy also promotes bodily functions, such as reducing pain and improving memory and concentration. Many types of essential oils used in aromatherapy have therapeutic functions for the body, mind, and spirit. Commonly used essential oils in aromatherapy include lavender, rose, geranium, and marjoram, with lavender being the most frequently used. All of these oils have antispasmodic and analgesic effects, making them beneficial for dysmenorrhea (88).

Furthermore, the effectiveness of aromatherapy essential oils may vary depending on the method of blending. By mixing oils together, a synergistic effect can be achieved (89). Therefore, it is necessary to determine standardized protocols through research on the selection of oils, their blending ratios, and the methods and duration of application. Additionally, the effectiveness of aromatherapy in antiviral, antimicrobial, and anti-inflammatory actions has been proven. Inhalation of aromatherapy can stimulate the olfactory system, increase parasympathetic nervous activity, reduce sympathetic nervous activity, and release neurotransmitters (such as endorphins), thereby effectively relieving pain and anxiety.

A study by Bakhtshirin F et al. (90) confirmed that, compared to placebo massage, lavender massage significantly reduced the VAS pain score. Statistically, lavender massage had a greater impact on the severity of primary dysmenorrhea than placebo massage. Raisi Dehkordi Z et al. (91) Conducted an experimental clinical trial with 96 female students living in dormitories at Tehran University of Medical Sciences in 2011. This study showed that inhaling lavender was effective in alleviating dysmenorrhea symptoms and could be safely used, as it had no side effects for all patients, being simple and cost-effective. However, there seems to be high heterogeneity among these studies, likely due to differences in the duration and timing of aromatherapy applications. Therefore, future research should compare the optimal application methods, cycles, and durations of aromatherapy. Studies must be rigorous, employing repeated research on the oils used during the intervention and experimental treatment periods, as well as strict control of external variables and study design, using standardized protocols to improve the quality of such evidence.

#### 2.2.5 Exercise

Exercise is widely used as a way to alleviate daily stress and control chemical changes in the immune system. Previous studies have shown that increasing progesterone levels through exercise can reduce the production of prostaglandins and pro-inflammatory cytokines, thereby decreasing pain (92, 93). Further research has indicated that exercise can reduce stress by decreasing sympathetic nervous system activity and increasing parasympathetic nervous system activity during rest, while also alleviating menstrual symptoms. Dysmenorrhea may be caused by increased uterine muscle contractions, which are mediated by the sympathetic nervous system (94). Stress contributes to increased sympathetic activity and may intensify uterine contractions, leading to greater pain during menstruation. Exercise that relieves stress can reduce sympathetic nervous system activity, thereby reducing menstrual symptoms.

Beta-endorphins, which act as natural painkillers, are released during exercise. Chantler et al. (95) suggested that exercise reduces the severity and duration of dysmenorrhea due to the release of endorphins, relaxation, stress relief, and improved blood flow. Mohammadi et al. concluded that regular and continuous aerobic exercise can control primary dysmenorrhea and heavy menstrual bleeding. Research by Aganoff and Boyle (96) indicated that regular aerobic exercise can improve mood and promote physical relaxation. Yoga is recommended as a generally safe intervention with almost no adverse side effects when practiced under the guidance of a trained instructor and has been proven to effectively alleviate primary dysmenorrhea.

Yoga not only stimulates the release of beta-endorphins but may also promote physical and mental health by downregulating the hypothalamic-pituitary-adrenal axis and the sympathetic nervous system. This effect has been shown to reverse the negative impact of stress on the immune system, reduce inflammatory markers, and lower stress, anxiety, heart rate, blood pressure, depression, and insomnia. Increasing evidence suggests that yoga can also improve quality of life by reducing pain through the release of pain-relieving endorphins. Low-quality evidence indicates that exercising for approximately 45–60 min, three times a week or more, regardless of intensity, may significantly reduce dysmenorrhea intensity by about 25 mm on a 100 mm VAS scale (97). Therefore, exercise is certainly effective in reducing the pain intensity of primary dysmenorrhea.

### 2.3 Lifestyle

**Figure 3:**
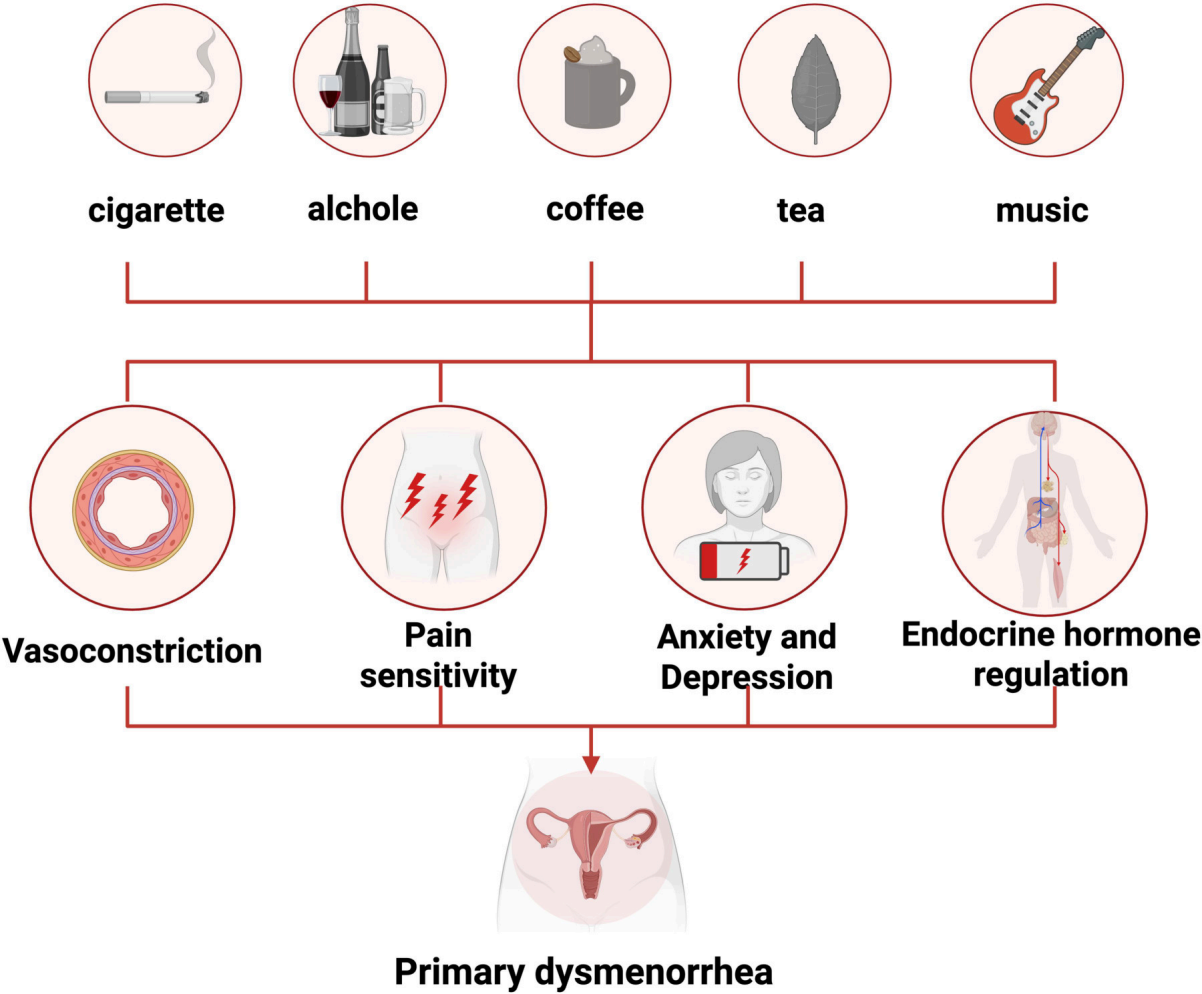
Lifestyle therapy for the treatment of primary dysmenorrhea.

A healthy lifestyle, including proper nutrition, exercise, not smoking, low alcohol consumption, controlled caffeine intake, and listening to music, can help alleviate dysmenorrhea symptoms (98, 99). While the genetic predisposition to dysmenorrhea cannot be changed, lifestyle choices can be adjusted to reduce the severity of the symptoms. Figure 3 depicts lifestyle therapy strategies for the treatment of primary dysmenorrhea.

#### 2.3.1 Smoking

The association between smoking and dysmenorrhea is well-known, involving various physiological and psychosocial factors that can exacerbate menstrual pain. On one hand, smoking causes vasoconstriction, reducing blood flow to the uterus, and increases menstrual pain by enhancing uterine muscle contractions and reducing oxygen delivery (100). On the other hand, smoking directly affects the endocrine regulation of menstruation, leading to an increase in prostaglandins, which are associated with inflammation and pain, potentially worsening dysmenorrhea. Additionally, smoking may disrupt estrogen metabolism, resulting in menstrual irregularities and increased pain (101). This is related to the anti-estrogenic extra-ovarian effects of smoking, which alter estrogen binding through serum proteins or estrogen receptors, enhance the metabolism of exogenous estrogen, or reduce the conversion of circulating androgens into estrogen, thereby affecting estrogen activity (102). Furthermore, since ovarian atrophy has been observed in animals exposed to smoke, smoking may have a direct toxic effect on the ovaries, although the reversible effects observed after smoking cessation do not fully support this.

Finally, from a psychosocial perspective, smoking as a coping mechanism for stress profoundly impacts menstrual health (103, 104). Stress itself often amplifies menstrual discomfort. Moreover, smokers’ health awareness and behaviors can influence their pain perception and management methods, affecting the severity of dysmenorrhea (105, 106).

#### 2.3.2 Drinking tea

Epigallocatechin-3-gallate (EGCG) in green tea can limit the biosynthesis of prostaglandins, such as prostaglandin E2 (PGE2) (107). Currently, non-steroidal anti-inflammatory drugs (NSAIDs) target the COX pathway to reduce the accumulation of prostaglandins, thereby decreasing pain intensity. EGCG provides a natural alternative to NSAIDs, which may act through similar mechanisms. In a study on chronic inflammation and its association with cancer, it was found that EGCG can limit the synthesis of PGE2 in acellular and human whole blood assays, possibly by inhibiting microsomal prostaglandin E synthase-1 (mPGES-1), an enzyme important in catalyzing the COX pathway (108). This conclusion was further validated in subsequent studies on human monocytes (109). Overall, these preliminary data suggest that EGCG can modulate prostaglandin synthesis through the COX pathway, potentially alleviating dysmenorrhea in women.

Additionally, drinking thyme tea can reduce the production of prostaglandins (PGs) by inhibiting the COX-2 pathway (110) and prevent oxidative stress by clearing lipid peroxide free radicals (oxidative stress index) in women with dysmenorrhea. Several studies have shown that women with dysmenorrhea have higher levels of lipid peroxides compared to normal women. Excessive production and release of PGs, especially PGF2*α*, in the endometrium lead to excessive activation of the endometrium and subsequent uterine hypoxia and ischemia (111). This tissue damage in the uterus activates phospholipase A2, which hydrolyzes cell membrane phospholipids, further spreading lipid peroxide free radicals and the production of arachidonic acid (a precursor for prostaglandin synthesis), worsening the severity of primary dysmenorrhea. The phenolic compounds in thyme leaves may inhibit the activity of phospholipase A2 and the subsequent arachidonic acid pathway (112, 113).

#### 2.3.3 Drinking alcohol

Alcohol has a significant impact on the hormonal balance in women, which is crucial for regulating the menstrual cycle. The disruption of hormonal levels caused by alcohol can exacerbate dysmenorrhea. In addition, alcohol stimulates the release of vasopressin, a hormone that constricts blood vessels, which may increase menstrual pain. Furthermore, alcohol is a diuretic, potentially leading to dehydration, which can worsen dysmenorrhea. Alcohol also lowers magnesium levels in the body, and magnesium is known to help relieve muscle spasms. Additionally, alcohol enhances the body's inflammatory response, which could intensify dysmenorrhea symptoms (114, 115).There is also evidence suggesting that alcohol may directly affect uterine muscles, increasing the severity of dysmenorrhea. However, individual responses to alcohol vary greatly and are influenced by factors such as alcohol consumption levels, overall health, and genetic predisposition. Therefore, while smoking or drinking alcohol alone may not have a substantial impact on the severity of dysmenorrhea, using both simultaneously can increase the risk of experiencing more severe menstrual pain.

#### 2.3.4 Have coffee

Coffee, one of the most consumed beverages globally, has a controversial role in managing primary dysmenorrhea pain, with existing research showing conflicting results. Zeru A B et al. (116) observed a direct independent association between coffee consumption and primary dysmenorrhea in their final model. Compared to daily coffee drinkers, those who do not drink coffee or drink it infrequently (once a week or less) had an 88% and 86% lower likelihood of experiencing primary dysmenorrhea, respectively. Similarly, previous studies conducted in Ethiopia, Turkey (117), and Kuwait (110) have indicated that caffeinated beverages may increase the risk of primary dysmenorrhea.

Although the mechanism by which coffee exacerbates dysmenorrhea remains unclear, one possible explanation is the vasoconstrictive effect of caffeine, which reduces blood flow to the uterus, thus intensifying menstrual pain. However, coffee is one of the most widely consumed caffeinated beverages worldwide, and caffeine, a naturally occurring alkaloid (118), has been shown to help manage pain. Caffeine competitively binds to adenosine receptors, inhibiting their important role in pain modulation and signal transduction (119). Furthermore, decades of clinical research have evaluated and proven the adjuvant analgesic effect of caffeine (120), and many over-the-counter (OTC) pain relievers now include low doses of caffeine as an adjunct.

Research by Soler-Martínez R et al. (121) confirmed that coffee extracts could prevent neuropathic pain caused by spinal cord injury, with the major polyphenolic compounds in coffee reducing reflexive pain responses. For example, chlorogenic acid, the primary polyphenolic compound extracted from coffee, has anti-inflammatory properties, reducing oxidative stress and inflammatory mediators, thus alleviating pain (122).

The controversy likely arises because most of the current studies are small-sample, single-center observational trials. Future large-sample, multi-center clinical trials could enhance the credibility of the evidence. Additionally, further research into the underlying mechanisms is needed to address the gaps in the existing evidence.

#### 2.3.5 Listen to music

The American Music Therapy Association (AMTA) defines music listening as a step in music therapy, a method aimed at protecting, maintaining, and improving individual physical and mental health (123). Listening to music has various positive attributes, including stress reduction and arousal, pain relief, promoting relaxation by affecting heart rate, blood pressure, and the release of endorphins, balancing emotional states, and treating anxiety and depression. Therefore, it can be used to alleviate dysmenorrhea (124). These positive outcomes can be explained by the characteristics and physiological effects of music. According to Western music theory, music is defined as “the art of organizing sound,” and its features are divided into two categories: the physical characteristics of sound (volume, pitch, and waveform) and the characteristics of the composition (melody, harmony, rhythm, and instrumentation). Rhythm is defined by the time signature and beat, with the beat indicating the temporal quality of the musical piece. Using these terms allows researchers to objectively categorize music and standardize interventions.Although the literature does not provide clear results on reducing dysmenorrhea in terms of rhythm, volume, or decibels, it is noted that in pain management, the sound level should not reach noise levels (51–75 decibels) (125). Recent neuroscientific research suggests that music can reduce physiological arousal and pain that increase during stress. Listening to music is associated with reduced physiological arousal and pain, as evidenced by decreases in cortisol levels, heart rate, and blood pressure (126–130). Therefore, many studies have investigated the effectiveness of art therapy in pain and anxiety management across various sample groups. However, more data is needed to determine the impact of art therapy on pain, perceived stress, and menstrual symptoms in women with dysmenorrhea. Table 2 summarizes the key clinical trials on non-ingestion interventions for primary dysmenorrhea.

**Table 2:** Non-ingestion intervention.

Therapy	Research type	Number	Time	Effect/result
TENS				
	Randomized controlled trial (RCT) (58)	40	2022	TENS reduced pain by 54% compared to SHAM (10%).
Acupuncture				
	Randomized controlled trial (RCT) (141)	60	2018	Compared to the control group, all variables in the study group, including pain, dysmenorrhea, headache, dizziness, and Visual Analog Scale (VAS) scores, were significantly reduced.
	Randomized controlled trial (RCT) (142)	72	2022	The application of randomized controlled neuroimaging trials will provide objective and effective evidence on how acupuncture and moxibustion help alleviate dysmenorrhea.
	Randomized controlled trial (RCT) (143)	78	2024	Acupressure and wrist-ankle acupuncture provide immediate pain relief. AWA represents an effective and safe non-invasive physical therapy option that patients can self-manage to alleviate abdominal pain.
	Randomized controlled trial (RCT) (144)	501	2014	Compared to non-acupoint and unrelated acupuncture in patients with primary dysmenorrhea, acupuncture at specific acupoints produced statistically significant effects, but no significant clinical effect.
	Randomized controlled trial (RCT) (145)	194	2011	Acupuncture is more effective than no acupuncture in relieving pain from single-point acupuncture for dysmenorrhea, but no differences were found between acupoint acupuncture, unrelated acupoint acupuncture, and non-acupoint acupuncture.
	Randomized controlled trial (RCT) (146)	26	2019	Vertical and horizontal acupuncture at the Sanyinjiao (SP6) acupoint have immediate analgesic effects on primary dysmenorrhea. Appropriate acupuncture techniques can be applied based on the patient's tolerance.
Finger Pressure				
	Randomized controlled trial (RCT) (147)	56	2025	Acupressure is an effective method for relieving pain, menstrual symptoms, and enhancing comfort.
	Randomized controlled trial (RCT) (148)	91	2016	Ear acupressure therapy is an effective intervention for relieving abdominal pain, back pain, and primary dysmenorrhea in South Korean female high school students.
	Randomized controlled trial (RCT) (87)	92	2015	Acupressure can improve primary dysmenorrhea.
	Randomized controlled trial (RCT) (149)	267	2025	It has been confirmed that acupressure and massage are effective methods for reducing pain intensity and the severity of menstrual symptoms in patients with primary dysmenorrhea, but they do not affect their quality of life.
Aromatherapy				
	Randomized controlled trial (RCT) (90)	80	2015	Lavender massage has a greater impact on the severity of primary dysmenorrhea compared to placebo massage.
	Randomized controlled trial (RCT) (150)	200	2016	Using lavender aromatherapy for two months may be effective in reducing the severity of pain associated with primary dysmenorrhea.
	Randomized controlled trial (RCT) (151)	50	2015	Compared to massage therapy alone, aromatherapy massage can reduce the severity of primary dysmenorrhea.
	Randomized controlled trial (RCT) (152)	100	2016	Rose essential oil aromatherapy is a non-pharmacological treatment that, as an adjunct to traditional therapies, may help alleviate pain in patients with primary dysmenorrhea.
Exercise				
	Randomized controlled trial (RCT) (153)	60	2025	Compared to the control group, the yoga group showed a reduction in dysmenorrhea and symptom severity, along with a significant improvement in quality of life.
	Randomized controlled trial (RCT) (154)	70	2018	Aerobic exercise can improve primary dysmenorrhea. Therefore, aerobic exercise can be used as a treatment for primary dysmenorrhea.
	Randomized controlled trial (RCT) (155)	62	2018	Exercise and lifestyle changes can be used to improve the quality of life and body awareness in patients with primary dysmenorrhea, while also reducing pain intensity.
	Randomized controlled trial (RCT) (156)	60	2023	Yoga can serve as an effective intervention for alleviating dysmenorrhea in women with primary dysmenorrhea.
	Randomized controlled trial (RCT) (157)	28	2020	The exercise group showed a significant decrease in the severity of abdominal pain, the total MSQ score, as well as the subscale scores for negative outcomes and management strategies.
	Randomized controlled trial (RCT) (158)	30	2024	Group A (Pilates exercises) showed a significantly lower average VAS score compared to Group B (Swiss ball exercises).
	Randomized controlled trial (RCT) (159)	73	2023	There was no significant difference in pain improvement between the control group and the intervention group.
	Prospective cohort study (160)	A group of adults	2025	There is no difference in dysmenorrhea intensity between the types of low-level physical activity.
	Randomized controlled trial (RCT (161)	30	2023	A 12-week Pilates intervention improved dysmenorrhea, partly mediated by improvements in physical function and sleep quality.
	Randomized controlled trial (RCT) (162)	97	2022	When used together, massage and progressive relaxation exercises are more effective than when used individually in reducing menstrual symptoms.
	Randomized controlled trial (RCT)) (163)	34	2017	The yoga program may be a complementary treatment for primary dysmenorrhea (PD).
Lifestyle				
Smoking				
	Prospective cohort study (164)	9,067	2016	Smoking and early initiation of smoking are associated with an increased risk of chronic dysmenorrhea.
Drinking alcohol				
	Cross-sectional study (165)	8,567	2025	There is a significant association between the simultaneous use of alcohol and smoking and an increased risk of dysmenorrhea.
Drinking tea				
	Case-control study (116)	252	2020	Drinking thyme tea and consuming vegetables and fruits are associated with pain-relieving tendencies for primary dysmenorrhea.
Coffee				
	Cross-sectional study (166)	1,809	2024	Coffee intake is a protective factor for the severity of Parkinson's disease (PD).
	Cross-sectional study (167)	382	2023	Physical exercise, smoking, alcohol consumption, and coffee drinking are not related to the incidence of dysmenorrhea.
	Cross-sectional study (136)	660	2022	Smoking, frequent consumption of sugar, carbonated drinks, coffee, tea, and energy drinks are associated with severe dysmenorrhea. On the other hand, alcohol has a positive effect on dysmenorrhea.
	Cross-sectional study (110)	763	2019	In the multivariate analysis, coffee drinking (*p*-value = 0.004) is significantly associated with dysmenorrhea.
	Retrospective study (168)	423	2008	Increased coffee consumption is associated with a negative attitude toward primary dysmenorrhea.
Listening to music				
	Randomized controlled trial (RCT) (169)	64	2024	Music therapy can effectively and significantly reduce dysmenorrhea pain in nursing students suffering from dysmenorrhea.

## 3 Discussion

Complementary and Alternative Medicine (CAM) interventions for primary dysmenorrhea, including vitamins, herbal supplements, acupuncture, exercise, transcutaneous electrical nerve stimulation (TENS), shiatsu, and aromatherapy, are commonly used to improve pain in patients. Exercise is a highly effective and safe method to improve primary dysmenorrhea. Multiple systematic reviews confirm that regular aerobic exercise (such as brisk walking, running, and swimming), yoga, and Pilates can significantly reduce the intensity and duration of menstrual pain, with no adverse side effects. Diet improvements are also a highly safe way to regulate inflammation factors and hormone levels associated with dysmenorrhea at the root. Acupuncture is recommended by the World Health Organization (WHO) and many pain management guidelines. Numerous randomized controlled trials (RCTs) demonstrate that acupuncture can effectively alleviate primary dysmenorrhea pain and reduce the use of painkillers. However, acupuncture should be performed by a licensed practitioner using sterile, single-use needles. Acupuncture carries low risk, with occasional minor bruising or soreness on the skin. High-frequency TENS is a proven effective modality, often yielding results comparable to ibuprofen. It is a non-invasive therapy that is portable, but it should be avoided on the front of the neck, near the eyes, or on the abdomen of pregnant women. Shiatsu, similar in principle to acupuncture, does not require needles. It works by pressing specific acupressure points (such as Sanyinjiao and Zusanli) to regulate the flow of energy (Qi) and blood. Many studies show that self-administered shiatsu can effectively reduce pain and decrease painkiller use. This method is very safe, cost-free, and can be performed independently, but it requires learning the correct pressure points and techniques, and its effectiveness varies between individuals. The effectiveness of various herbal supplements in treating primary dysmenorrhea varies significantly. These supplements typically possess anti-inflammatory, antispasmodic, and hormone-regulating properties. However, the quality of herbal supplements on the market is uneven, with challenges related to standardization and potential herb-drug interactions (e.g., with anticoagulants). Therefore, it is recommended to use herbal supplements under the guidance of a doctor or pharmacist and to choose reputable brands. Vitamins, when taken at recommended doses, are very safe, though they have a slow onset of action. It typically takes 2–3 menstrual cycles to assess their effect. Vitamins participate in neuroregulation, hormone balance, and antioxidant processes. Existing evidence supports their effectiveness as an adjunctive treatment, but further research is needed. Most aromatherapy studies support the use of lavender and rose essential oils, through inhalation or abdominal massage, for relieving dysmenorrhea. This method is easy to use and promotes relaxation, but its effects are subjective and may cause allergic reactions.

In summary, non-pharmacological management of primary dysmenorrhea is a multi-layered, individualized approach. Starting with fundamental lifestyle changes and then adding other safe and effective therapies based on individual responses and preferences is the best way to control dysmenorrhea and reduce dependence on painkillers. Focusing on exercise and diet, combined with TENS and acupuncture during menstruation to manage acute pain, along with self-administered shiatsu for additional pain relief, is an effective strategy. Vitamin supplementation and herbal supplements should be used under the guidance of professionals. Aromatherapy can be considered when stress and anxiety exacerbate dysmenorrhea.

Non-steroidal anti-inflammatory drugs (NSAIDs) and hormone therapy are the first-line treatment options recommended in global gynecology guidelines, supported by thousands of randomized controlled trials and years of clinical experience for their effectiveness and safety. NSAIDs are commonly associated with gastrointestinal discomfort (e.g., heartburn, nausea), and long-term or high-dose use can lead to gastric ulcers, kidney damage, and cardiovascular risks. Their effects are also limited for some individuals. Hormone therapy can cause breakthrough bleeding, mood swings, breast tenderness, weight gain, and carries a small risk of venous thromboembolism. It is not suitable for all women, such as smokers or those with a history of thrombosis. NSAIDs do not address the root cause of dysmenorrhea, as they only relieve symptoms during pain episodes and cannot prevent future occurrences of dysmenorrhea. Many complementary and alternative medicine (CAM) methods, such as exercise and dietary adjustments, aim to improve overall health rather than just addressing a single symptom. These approaches are often gentler than medications, with good body tolerance and minimal side effects. Patients can actively participate in managing their health, such as through regular exercise, dietary changes, or using TENS devices. This “sense of self-efficacy” itself has a positive impact on pain relief. For women who prefer or are unable to use medications (e.g., due to allergies to NSAIDs or contraindications), CAM offers valuable alternative options. Moreover, the evidence supporting some CAM approaches, especially acupuncture, regular exercise, and certain herbs (e.g., ginger), is strengthening. A growing body of medium- and high-quality research has confirmed their effectiveness, and these methods are gradually being integrated into some mainstream medical guidelines.

Not all CAM interventions are effective and safe. Not all CAM methods are supported by the same level of scientific evidence, and their effects can vary from person to person. Unlike taking a single ibuprofen for quick pain relief, many CAM methods (such as dietary changes, vitamin supplementation, and regular exercise) require weeks or even months to show results. In particular, herbal supplements can vary greatly between brands in terms of purity, potency, and dosage, and may contain contaminants or undeclared ingredients. The regulation of herbal supplements is not as strict as that for pharmaceuticals. For therapies like acupuncture, it is essential to find qualified professionals, and the costs can be relatively high. Using supplements on your own may pose risks of drug interactions (such as some herbs interacting with anticoagulant medications). Therefore, it is important for individuals to approach CAM interventions with caution, ensuring proper guidance from healthcare professionals and being aware of potential risks.

For women, it is important to understand the risks and benefits of CAM for primary dysmenorrhea. Although the trends in CAM research are largely positive, more work is needed to better understand their effectiveness and implementation. The evidence regarding the effectiveness of CAM therapies is promising, but these studies are often hindered by small sample sizes and/or being limited to a single ethnicity, making it unclear whether these treatments are effective across all races and nationalities. Various therapies show mixed results. Most studies rely on subjective symptom ratings, with some objective findings indicating potential benefits. Further investigation is needed to determine the true benefits of these products. Given the minimal risks associated with CAM therapies, integrating these modalities into standard care will continue to require additional research to assess their pharmacoeconomic value while maintaining low adverse effects, alongside improving quality of life. Therefore, before making any clinical recommendations, larger-scale studies involving adolescent participants from diverse racial backgrounds are necessary.

It is important to note that mainstream methods and complementary alternative therapies (CAM) are not opposites, but rather can be complementary tools. The wisest approach is to combine the strengths of both: leveraging the efficacy and speed of mainstream methods, while incorporating the holistic and safe aspects of CAM. Under the guidance of a professional doctor, a personalized dysmenorrhea management plan can be created, tailored to an individual's unique needs and circumstances.

Future research on CAM should include control groups, validated instruments, standardized protocols, and appropriate follow-up measures to assess the maintenance of effects, ensuring rigorous study designs. Such studies will likely help the field understand how to best apply CAM in clinical settings and provide recommendations for its use in clinical practice. However, it is important to note that larger trials are needed to further support the available evidence before widespread implementation. Nevertheless, given the relatively low risks of these modalities, clinicians should consider these potential adjunctive treatments for patients with refractory symptoms.
